# Supplementary material for: Coenzyme Q10 encapsulated in micelles ameliorates osteoarthritis by inhibiting inflammatory cell death
Source: PLoS One. 2022 Jun 24;17(6):e0270351. doi: 10.1371/journal.pone.0270351 (PMC9231733; doi:10.1371/journal.pone.0270351)
Supplement: S1 File — (DOCX) [file pone.0270351.s001.docx]

**Coenzyme Q10 encapsulated in micelles ameliorates osteoarthritis by inhibiting inflammatory cell death**

Hyun Sik Na^1, 2, *^, Jin Seok Woo^1, *^, Ju Hwan Kim^3, *^, Jeong Su Lee^1,2^, In Gyu Um^1,2^, Keun-Hyung Cho^1,2^, Ga Hyeon Kim^3^, Mi-La Cho^1, 2, 4, #^, Sang J. Chung^5, #^, Sung-Hwan Park^1, 6, #^

^1^The Rheumatism Research Center, Catholic Research Institute of Medical Science, College of Medicine, The Catholic University of Korea, Seoul 06591, Korea, ^2^Department of Biomedicine & Health Sciences, College of Medicine, The Catholic University of Korea, Seoul 06591, Korea, ^3^AbTis Co. Ltd., Suwon 16648, Korea, ^4^Department of Medical Lifesciences, College of Medicine, The Catholic University of Korea, Seoul 06591, Korea, ^5^Department of Biopharmaceutical Convergence, School of Pharmacy, Sungkyunkwankwan University, Suwon 16419, Korea, ^6^Division of Rheumatology, Department of Internal Medicine, Seoul St. Mary's Hospital, College of Medicine, The Catholic University of Korea, Seoul 06591, Korea

* These authors contributed equally to this work.

# Authors to whom correspondence should be addressed. Email: iammila@catholic.ac.kr (M.L.C.), sjchung@skku.edu (S.J.C.), and rapark@catholic.ac.kr (S.H.P.)

**Supplementary Table 1. List of primers for real-time PCR in this study**

| **Gene Name** | **Forward Primer** | **Reverse Primer** |
| --- | --- | --- |
| **β-actin** | CATGTACGTTGCTATCCAGGC | CTCCTTAATGTCACGCACGAT |
| **MMP-1** | CTGAAGGTGATGAAGCAGCC | AGTCCAAGAGAATGGCCGAG |
| **MMP-3** | CTCACAGACCTGACTCGGTT | CACGCCTGAAGGAAGAGATG |
| **MMP-13** | CTATGGTCCAGGAGATGAAG | AGAGTCTTGCCTGTATCCTC |
| **RIPK1** | GACGAAGCCAACTACCATCTT | TCTCCTTTCCTCCTCTCTGTT |
| **RIPK3** | ATGTCGTGCGTCAAGTTATGG | CGTAGCCCCACTTCCTATGTTG |

**Supplementary Table 2. Quantification analysis of nanoemulsion by HPLC**

| Component | Initial concentration  (mg/mL) | Final concentration  (mg/mL) | % |
| --- | --- | --- | --- |
| EPA | 1.0 | 0.24 | 24 |
| Coenzyme Q10 | 1.0 | 1.0 | 100 |
| Glycyrrhizinate | 1.0 | 0.6 | 60 |
