# Supplementary material for: Coenzyme Q10 encapsulated in micelles ameliorates osteoarthritis by inhibiting inflammatory cell death
Source: PLoS One. 2022 Jun 24;17(6):e0270351. doi: 10.1371/journal.pone.0270351 (PMC9231733; doi:10.1371/journal.pone.0270351)
Supplement: Attachment — Submitted filename: Na et al_Response to Reviewers.docx [file pone.0270351.s003.docx]

Reviewer #1: This manuscript demonstrates that delivery of Co-enzyme Q10 in micelles can alleviate pain, inflammatory cell death and tissue destruction in a rat model of osteoarthritis. In addition, they demonstrate that in vitro treatment Co-Q10 treatment attenuates the inflammatory cell death in OA patient chondrocytes induced by IL-1B.

There are issues that need to be addressed.

: We appreciate the reviewer for her/his enthusiasm about our manuscript. Please see our detailed response to your comments below.

1. This manuscript does not provide new mechanisms of Co-enzyme Q10 in OA from what they published previously.

: Thank you pointing this out. We have done this experiment with CoQ10 alone and we saw better effect of CoQ10-micelle than CoQ10 alone. We replaced figures, please see figure 1, 2, 3 in revised manuscript.

2. The authors state that micelles can increase the potency of drug delivery, this is not directly assessed in this manuscript. Administration of CoQ10 as a control would be needed to determine this.

: We totally agree with reviewer’s comment. As we said above, we have done this experiment before. Please see revised figure 1, 2, 3.

3. They have used Celebrex as a positive treatment control, but it is unclear what they negative control mice are being given. Are they administered empty micelles?

: We totally agree with reviewer’s comment. We have done this experiment before with micelle alone, too. We did not see any effect of micelle alone. Please see revised figure 1, 2, 3.

4. In figure 5. It is unclear how the qPCR data was quantified for each gene of interest? Were these delta-delta Ct values or was the data relative RNA compared to a reference gene expression?

: We apologize for confusing you. We used delta-delta Ct value, but it was not able to see detailed value because value of control was too small. We put new bar graph with broken y axis. Please see revised figure 7.

5. The primer sequences used should be provided.

: We added the information of primer sequences in table 1. Please see methods section and supplementary information file.

Reviewer #2: With this work, the authors intend to demonstrate that micellized CoenzymeQ10 has a protective role on the OA inflammatory and catabolic features of the disease, using in the study a rat model of OA.

In general, the authors provide extensive several parameters that ensure the quality of the results obtained. The abstract and introduction is, in general written, in clear English with few flaws.
Nevertheless within the next sections Ia have serious concerns that should be clarified.

: We appreciate the reviewer for her/his enthusiasm about our manuscript. Please see our detailed response to your comments below.

Materials and methods section:

Induction of osteoarthritis and treatment with CoQ10-micelles:

Suggestion: Please describe how many animals were considered for each group and how long after treatment with MIA were animals considered OA. Please describe also how long was the treatment with coQ10.

: We apologize for confusing you. We described detailed protocol in method section. Please see revised manuscript.

Results Section:

It would have been nice to see the improvement/decrease results expressed in concrete values of mean+/- SEM.

Legend of figure 2 needs improvement since it is not easy to understand what is stated in special in what refers to the timepoints in which the measures were taken.

: We apologize for confusing you. We replaced the legend of figure 2. Please see revised manuscript.

Please explain in detail how you determined these areas of positivity for cytokines and MMP, in order to clarify the results presented, since the representative images are difficult to visualize in the quality presented.

: We apologize for confusing you. In IHC images, brown dots mean positive of indicated genes. We replaced figure with better quality images. Please see revised figure 5, 6.

Figure 6 and its legend do not correspond. Probably by mistake authors presented in figure 6 the same panels of representative immunuhistochemstry images for Cytokines and MMP from figure 5 and not representative images of immunohistochemical staining of RIP1, RIP3, and pMLKL as stated.
: We apologize for confusing you. It has been rectified. Please see revised figure.

Since the effect of coQ10 in OA was already reported it would have been interesting to see if the levels of micellized co-Q10 would have increased the levels of the freed coQ10 in the blood samples/synovial fluid of the animals in study.

: Thank you pointing this out. We have done this experiment with other groups include CoQ10 alone and micelle alone. And we found that CoQ10-micelle show better therapeutic effect than CoQ10 alone, and micelle alone did not show any therapeutic effect. We put these result in figure 1, 2, 3. Please see revised figures.

Although the English used is grammatically correct the text is sometimes confusing, especially in the discussion section.

Please reformulate the second paragraph of the discussion section.

E.g: “Increased MMP levels cause inflammatory diseases such as OA”, please explain.
: Thank you pointing this out. We replace the sentence. Please see revised manuscript.

The discussion fails to bridge more often between the results obtained by the authors in this model and with micellized co-Q10, and what is already known from previous studies with Q10 and OA. They also fail to mention at any point why human chondrocytes are used in the process and that the entire study was performed in a rat model.

In my opinion, although interesting, the study does not provide significant novelty and needs some revision before it can be published.

: Thank you for your comments. We added mention about relationship between CoQ10 and OA. In this study, we examined the therapeutic effect of CoQ10-micellee in OA animal model. At the same time, we would like to confirm the therapeutic potential of CoQ10 in human object. With this reason, we used human chondrocytes. We mentioned about that in discussion section. Please see discussion section in revised manuscript.
